# Subtalar dislocation: a narrative review

**DOI:** 10.1007/s12306-022-00746-x

**Published:** 2022-04-18

**Authors:** G. Lugani, M. Rigoni, L. Puddu, A. Santandrea, F. Perusi, D. Mercurio, F. Cont, B. Magnan, F. Cortese

**Affiliations:** 1 Department of Orthopaedics and Traumatology, University of Verona, Piazzale Aristide Stefani 1, 37126 Verona, Italy; 2 Department of Orthopaedics and Traumatology, Rovereto Hospital, Rovereto, Italy

**Keywords:** Subtalar dislocation, Lateral subtalar dislocation, Foot and ankle trauma, Irreducible dislocation, Acquired clubfoot, Acquired flatfoot

## Abstract

**Background:**

Subtalar joint dislocation (1% of all dislocations) is the permanent loss of articular relationships in the talonavicular and talocalcaneal joints, without other involvement of the foot. Dislocation can occur medially (85%), laterally (15%), posteriorly (2.5%) and anteriorly (1%). Reduction can be performed by closed or open technique; lateral dislocations often require open reduction because of inclusion of soft tissues or bone fragments. Lateral dislocations are frequently complicated by bone exposure, risk of infection and associated soft tissues injuries.

**Aim of the study:**

The aim of this study is to explain main characteristics and to clarify the most important pitfalls of subtalar dislocations.

**Materials and methods:**

We examined 47 articles published in the last thirty years (389 cases). For each dislocation we reviewed its main characteristics: direction, bone exposure, need for open reduction and for surgical stabilisation, associated injuries and method used for diagnosis.

**Results:**

Medial dislocations (68.1%) has greater incidence compared to lateral ones (27.7%). Bone exposure (44.5%), associated lesions (44.5%) and need for surgical reduction (48.2%) are much more represented in lateral dislocation than in the others.

**Conclusions:**

Subtalar dislocations, especially the lateral one, represent a challenge for surgeons. Lateral subtalar dislocation occurs following high-energy trauma often involving associated injuries. Closed reduction could be unsuccessful and patients must undergo surgical reduction. After reduction CT scan is recommended. Our narrative review confirms these findings.

## Introduction

Subtalar (or peritalar) dislocation means the simultaneous and permanent loss of articular relationships in the talonavicular and talocalcaneal joints, without fracture of the talar neck and without tibiotarsal or calcaneocuboid joint involvement.

Described for the first time in 1811 by Judcy and DauFaurest [1], it is a very rare dislocation (1–2% of all dislocations) [2].

Broca described this type of dislocation for the first time and classified it by subdividing it into medial, lateral or posterior forms, based on the position of the foot in relation to the talus [3]. Malaigne and Burger supplemented this classification through the addition of anterior dislocation [4].

Subtalar dislocation occurs most frequently in a medial direction (85%) and less frequently laterally (15%–20%), while posterior (2.5%) and anterior (1%) dislocations are exceptions [5]. In lateral dislocations, the head of the talus is dislocated medially, while the remainder of the foot remains lateral. In medial dislocations, the inverse occurs [6].

Subtalar dislocation primarily affects active young male adults (M:F—6:1), and is frequently associated with fractures of the hindfoot, midfoot and ankle.

Medial dislocation, which is also described as “acquired clubfoot”, is caused by distortive trauma due to forced inversion with the foot in plantar flexed position. The consequent stress to the lateral perimalleolar ligament apparatus causes the rupture of the talocalcaneal and talonavicular ligaments, so that the talus remains correctly positioned in the tibiotarsal joint while the subtalar dislocation occurs.

The term “acquired flatfoot” refers to lateral subtalar dislocation, the second most frequent pattern [7]. In this case, the forces act by stressing the medial side of the foot, causing a forced eversion injury. High-energy trauma is required to cause a lateral subtalar dislocation, which explains the high frequency of associated problems such as fractures or soft tissue injuries (described in the literature with an incidence of up to 40%) [8] and bone exposure. Lateral dislocations are known to be the most difficult to reduce because of the frequent inclusion of soft tissues (such as the posterior tibial tendon, the flexor digitorum longus or the joint capsule) or bone fragments and require surgical reduction in almost all cases. The outcome of lateral dislocations is often less than satisfactory, due to the high frequency of bone exposure and associated injuries [9].

We propose a narrative review of the literature on subtalar dislocations of the last thirty years; the aim of this study is to explain main characteristics and to clarify the most important pitfalls of subtalar dislocations, especially in the more difficult treatment of the lateral ones.

## Material and methods

The search for articles was carried out in Pubmed, Scopus, DARE, Proquest and Google Scholar databases using a combination of the following key words: “Subtalar dislocation” and “Lateral”, “Subtalar dislocation” and “Medial”, “Subtalar dislocations” and “Open reduction”, “Subtalar dislocation” and “Lesion”, “Talus” and “Lateral process”.

We searched for articles of the types “case report”, “case series” and “review”.

We considered articles published in the last 30 years, drafted in English.

We considered articles to be suitable where they provided complete information about the following characteristics: direction of the dislocation, bone exposure, any need for open reduction, use of K-wires or external fixators for stabilisation, use of CT check-ups post-reduction, and presence of associated injuries.

Articles which not met these inclusion criteria was eliminated from the review.

Each article meeting the inclusion criteria was analysed critically by two independent reviewers to its methodological quality. In the case of a discrepancy between the assessments made by the reviewers, a third reviewer was involved to resolve the dispute.

## Results

Our review considered 47 articles (23 case reports, 17 case series, four reviews and three case reports and reviews), published between 1991 and 2018, covering a total of 389 patients (Table 1).

**Table 1:** Articles published between 1991 and 2018 about subtalar dislocation of the foot

Revised articles			Dislocation							Reduction		
Authors	Year	Cases	M	L	P	A	Closed	Open	Associated lesions	Open	K wires	TC post
Azarkane et al. [10]	2014	1	1				1					
Bak et al. [11]	1991	1	1				1		1			
Bali et al. [12]	2011	1			1		1					1
Banerjee et al. [13]	2017	1		1			1		1			1
Bibbo et al. [9]	2001	9	7				7		7	1		7
				2			2		2			2
Bibbo et al. [14]	2003	25	15				9	6	22	4		
				8			9	1		4		
Camarda et al. [15]	2009	1			1		1					1
Camarda et al. [16]	2015	13	9				9		2	1		1
				3			3		2		1	1
					1		1					
Chuo et al. [17]	2005	1				1	1		1	1	1	
de Palma et al. [18]	2007	30	20				20					
				10			10					
de Palma et al. [19]	2008	3	1				1			1		1
				2			2			2		2
Dimentberg et al. [20]	1995	5	5				4	1	5	1		
Edmunds et al. [21]	1991	10	5					5	5	5	5	
				4				4	1	4		
					1			1	1	1		
Fotiadis et al. [22]	2009	1	1				1		1			1
Gaba et al. [23]	2017	1			1		1					1
Garofalo et al. [24]	2004	18	13				11	2	3		2	
				5			1	4	5		2	
Giuffrida et al. [25]	2003	6	6				6		6	3		
Ghani et al. [26]	2014	1		1				1	1			1
Giannoulis et al. [27]	2015	1	1				1					1
Goldner et al. [8]	1995	15	5					5	5	5	3	
				10				10	10	10	4	
Hoexum et al. [28]	2014	2	2				2		2	1	1	1
Hui et al. [29]	2016	1				1	1		1			1
Inokuchi et al. [30]	1997	20	13				12	1	7	1		
				4			4		4	2		
					2		1	1	1	1		
						1	1		1			
Jayaprakash et al. [31]	2011	1		1				1		1	1	
Jerome et al. [32]	2007	1	1				1					1
Jerome et al. [33]	2008	1		1			1					1
Jerome et al. [34]	2008	1		1				1				1
Jungbluth et al. [35]	2010	23	16				11	5		6	5	
				6			4	2		3	5	
					1		1					
Kanda et al. [36]	2001	1				1	1		1			1
Karampinas et al. [37]	2009	9		9				9		9	9	
Kinik et al. [38]	1999	1	1				1					1
Krishan et al. [39]	2003	1			1		1					
Kulambi et al. [40]	2014	1		1				1		1	1	
Lasanianos et al. [41]	2011	8	8				7	1				
McKeag et al. [42]	2015	1	1				1		1			1
Milenkovic et al. [43]	2006	11	9					9	5	9		
				2				2	2	2		
Merchan et al. [44]	1992	39	29				15	14	18	15	3	
				10			8	2	5	1	1	
Perugia et al. [2]	2002	45	37				37					
				8			8					
Ruhlmann et al. [45]	2016	13	10				10					2
				3			3					2
Spechullli et al. [46]	2007	14	11				6	5	5	5		
				3				3	3	3		
Stafford et al. [47]	2013	1	1				1					1
Tucker et al. [48]	1998	1		1			1		1			
Valdivieso et al. [49]	1996	19	16				12	4	11	6		
				3				3	2	3		
Veltman et al. [50]	2016	1		1				1	1	1	1	1
Wagner et al. [51]	2004	27	19				19		9	2	7	19
				7			7		7	6		7
						1		1	1	1	1	1
Yglesias et al. [52]	2018	1	1				1		1	1	1	
Zaraa et al. [53]	2016	1		1				1	1	1	1	1

M—Medial; L—Lateral; P—Posterior; A—Anterior

The results described below are shown in Table 2 (Table 2).

**Table 2:** Results of our review of 389 patients with subtalar dislocation

Subtalar dislocation of the foot Review of 389 patient		Dislocation			Reduction		
		Closed	Open	Associated lesions	Closed	Open	K wires
Medial	265 68.1%	207 78.1%	58 21.9%	94 35.5%	197 74.6%	67 25.4	27 10.2%
Lateral	108 27.7%	64 58.2%	46 41.8%	48 44.5%	57 51.8%	53 48.2%	26 23.6%
Posterior	9 2.3%	7 77.8%	2 22.2%	2 22.2%	7 77.8%	2 22.2%	0 0%
Anterior	5 1.3%	4 80%	1 20%	5 100%	3 60%	2 40%	2 40%
Total	387^a^	282 72.5%	107 27.5%	171^b^ 44%	264^c^ 68%	124^c^ 32%	55 14.2%

M—Medial; L—Lateral; P—Posterior; A—Anterior

^a^Missed information about direction of dislocation in 2 patients of the article of Bibbo et al. [14]

^b^Missed information about correspondence between direction of dislocation and presence of associated lesions in 22 patients of the article of Bibbo et al. [14]

^c^Missed information about method of reduction in 1 patient of the article of Dimentberg et al. [20]

The direction of dislocation was specified in 387 patients out of 389: this showed 265 medial dislocations (68.1%), 108 lateral (27.7%), 9 posterior (2.3%) and 5 anterior (1.3%). In the article by Bibbo et al. [14], the direction of dislocation was not stated for two of the 25 patients because no data could be found in the clinical records.

Dislocations complicated by bone exposure occurred in 107 of 389 patients (27.5%), compared to 282 closed dislocations (72.5%).

The ratio of closed and exposed dislocations is indicatively maintained among medial dislocations (closed: 207–78.1%; exposed: 58–21.9%), posterior (closed: 7–77.8%; exposed: 2–22.2%) and anterior (closed: 4–80%; exposed: 1–20%); while for lateral dislocations, closed (64–58.2%) and exposed (46–41.8%) dislocations are more uniformly distributed.

Reduction in the dislocation was achieved using solely external intervention in 264 patients (68%), while open reduction was required in 124 patients (32%). In one of the five patients in the article by Dimentberg et al. [20], the type of reduction performed was not described.

Once again, this ratio of around 2:1 between closed and open reductions was approximately maintained among medial dislocations (closed: 197–74.6%; open: 67–25.4%), posterior (closed: 7–77.8%; open: 2–22.2%) and anterior (closed: 3–60%; open: 2–40%). In the case of lateral dislocations, however, it was necessary much more frequently to perform open reduction (closed: 57–51.8%; open: 53–48.2%).

To stabilise the reduction, K-wires or external fixators were used in 27 medial dislocations (10.2% of the total number of medial dislocations), 26 lateral (23.6%) and two anterior (40%), and thus a total of 55 cases (14.2% of the total number of dislocations). There were no cases of posterior dislocations that required final stabilisation using K-wires or external fixators.

Of the total of 389 patients, 171 (44%) were described as having one or more associated injuries. These injuries (fractures of the midfoot, hindfoot or ankle and tendon or vasculonervous injuries) were present in 35.5% of medial dislocations (94 cases), 44.5% of lateral dislocations (48 cases), 22.2% of posterior dislocations (two cases) and all five cases of anterior dislocation.

The article by Bibbo et al. [14] does not provide a description of the correspondence between associated injuries (found in 22 cases in 25 patients) and direction of dislocation.

A check-up CT following completion of the reduction manoeuvre was only performed in 63 patients, namely 16.2% of cases (14% of medial dislocations, 18.5% of lateral, 33.3% of posterior and 60% of anterior).

Associated injuries were diagnosed using conventional x-rays in 128 patients of 149 (85.9%) and CT scans in 19 patients of 149 (12.75%). In two cases (fracture of the head of the talus and injury to the posterior tibial tendon and flexor digitorum longus), diagnosis was made directly in the operating theatre. In 22 patients with associated injuries in the Bibbo et al. [14] case series for whom the direction of dislocation is not stated, no indication of the method (x-ray, CT scan) used to make the diagnosis has been provided.

## Discussion

The greater complexity of managing lateral dislocation is probably the reason why recent years have seen the attention of authors focused more on these dislocations than on medial forms. In fact, the prevalence of lateral dislocations in our review is almost 30%, compared to 15%–20% reported in the literature.

The purpose of this study is to highlight the most insidious aspects in the diagnosis and treatment of subtalar dislocations, especially the more complicated lateral dislocations.

Indeed, lateral dislocations are made even more challenging by the ease with which the head of the talus is exposed through the medial and skin capsular tissues in a context of high-energy trauma. The technical difficulties associated with reduction are thus increased by the need to ensure meticulous soft tissue handling to prevent insidious infectious complications. Our review confirms this fact: medial dislocations are exposed in 21.9% of cases, while lateral forms are exposed in 41.8%.

The reduction manoeuvre extensively described in the literature involves bending the knee to release the traction of the Achilles tendon on the calcaneus, followed first by accentuating and then inverting the deformity.

We documented a failure of closed reduction in 32% of dislocations. Difficulties in performing reductions are much more evident for lateral dislocations (48.2% of open reductions) than for medial forms (open reduction in 25.4% of cases).

The reduction in subtalar dislocations is often prevented by the inclusion of soft tissues or bone fragments.

In the case of lateral dislocations, inclusions most often involve the posterior tibial tendon, and sometimes the flexor digitorum longus or flexor hallucis longus tendon. In the latter case, it is typical to note the flexion of the first toe as an indirect sign of stretching of the flexor hallucis longus tendon.

In medial dislocations, instead, the extensor retinaculum constitutes the principal obstacle to closed reduction. Less frequently, open reduction is made necessary by the inclusion of the extensor hallucis brevis and the extensor digitorum brevis [54].

**Fig. 1:**
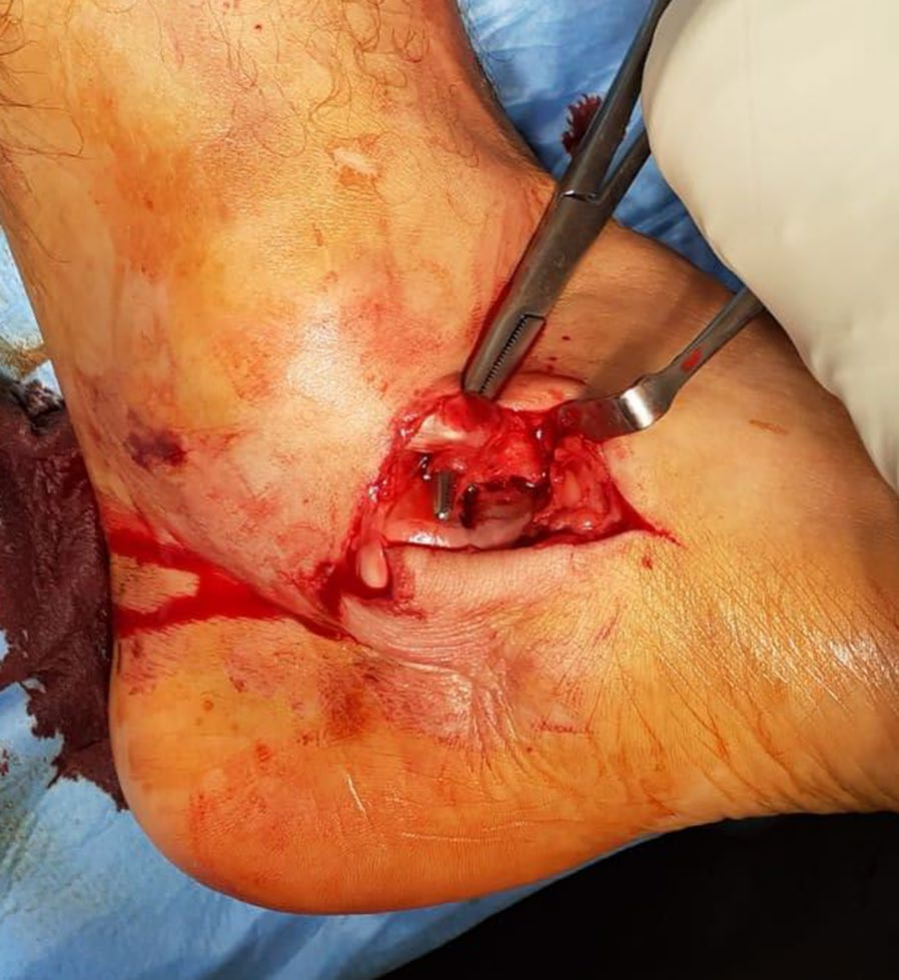
Inclusion of the posterior tibial tendon preventing closed reduction in the lateral subtalar dislocation

In dislocations complicated by inclusion of tendon or capsular structures or fracture fragments, reduction using external manoeuvres is impractical and it is absolutely necessary to carry out surgical reduction as soon as possible. This involves identifying the interposed structures and moving them away from the subtalar joint (Fig. 1). Awareness of this eventuality is essential in preventing futile attempts at reduction and enabling rapid repositioning of the talus, a factor that strongly influences the risk of avascular necrosis.

**Fig. 2:**
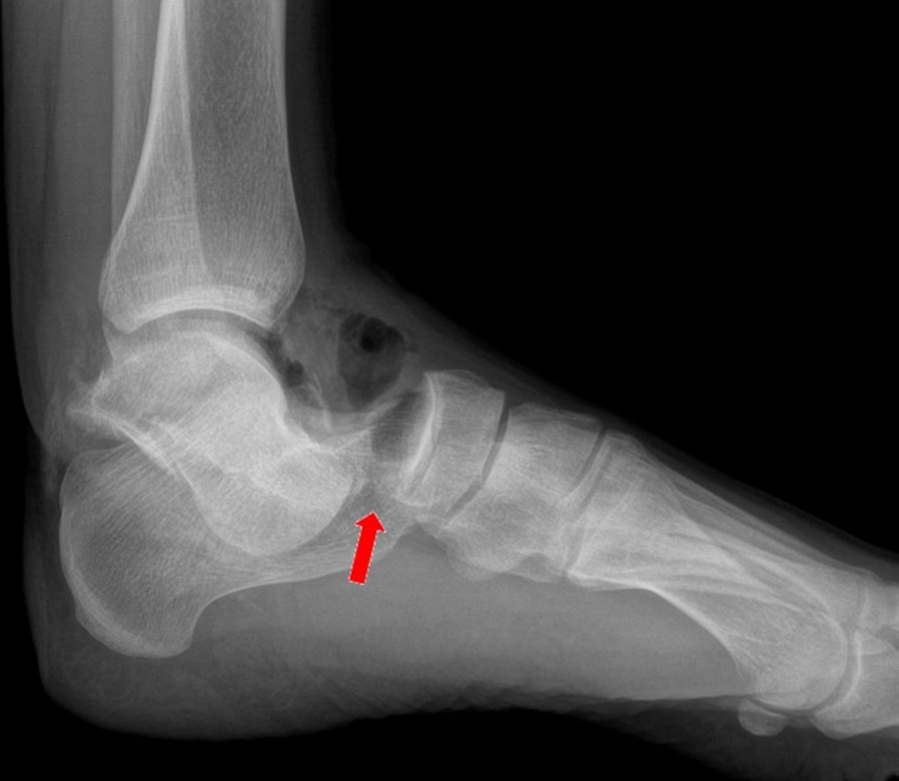
Latero-lateral X-ray showing a lateral subtalar dislocation. The red arrow shows inclusion of the posterior tibial tendon between the head of the talus and the navicular bone

With regard to the more complicated lateral dislocations, an x-ray indicator of the difficulty of closed reduction can be seen clearly on the latero-lateral views (Fig. 2, red arrow). As shown in Fig. 2 the empty space between the head of the talus and the tarsal navicular bone is due to the inclusion of the posterior tibial tendon.

**Fig. 3:**
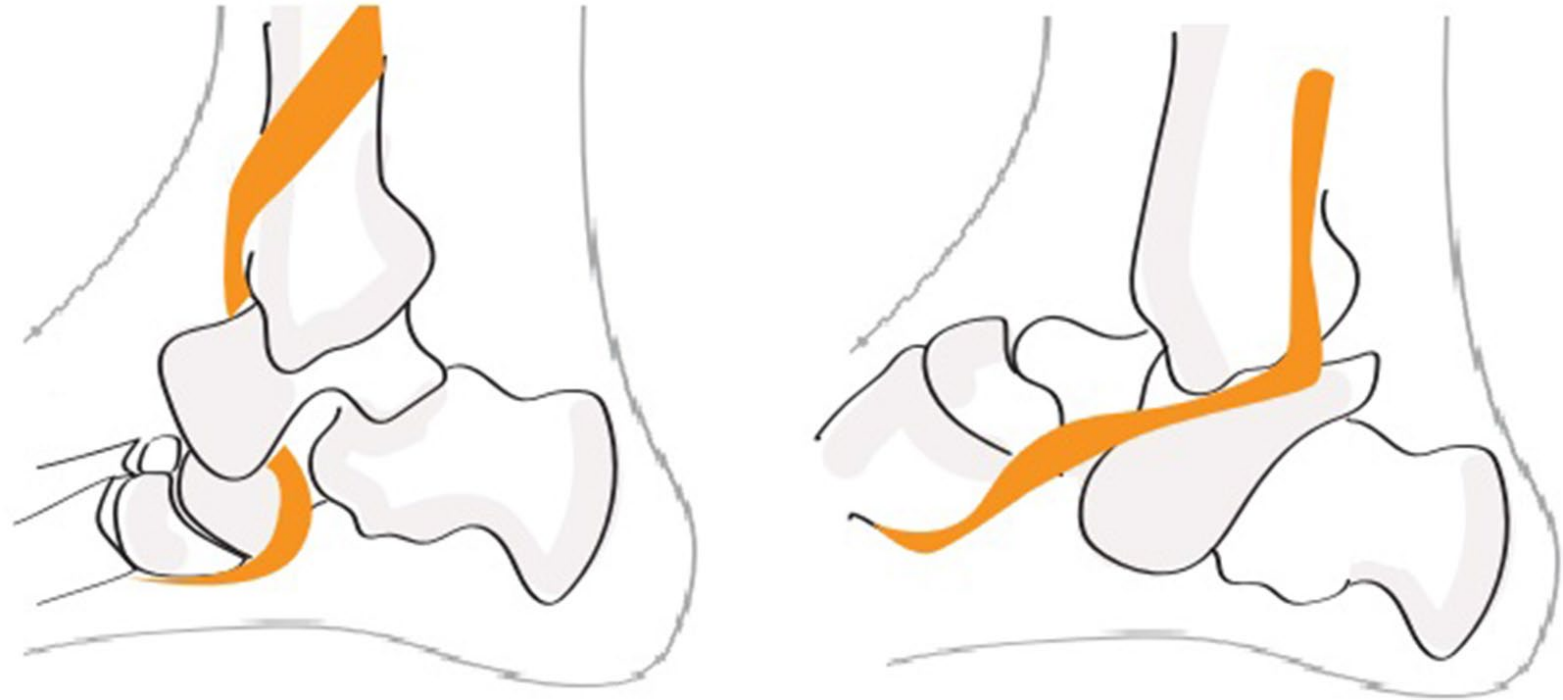
Principal methods for inclusion of the posterior tibial tendon in lateral subtalar dislocations [55]

Observation of this x-ray sign and an awareness of the main methods for inclusion of the posterior tibial tendon in lateral dislocations [55] (Fig. 3) can facilitate the reduction in lateral dislocations.

Once the reduction in the dislocation has been achieved, it is not sufficient to take simple x-rays. A CT scan should be performed to exclude misrecognised fractures that can lead to osteoarthritis and chronic instability, especially in the hindfoot [9, 56].

Of the total of 171 fractures associated with the 389 cases in our review, 19 (12.75%) were diagnosed using CT scans.

The fractures most commonly associated with this type of dislocation are those of the body, neck or head of the talus or of the sustentaculum tali or fractures of the lateral process of the talus, also known as a Snowboarder’s fracture. Fractures of the cuboid, navicular and calcaneus bones occur more rarely.

For this aspect also, we can confirm that lateral dislocations are more difficult to manage. According to our review, these forms have associated injuries in 44.5% of cases, compared to 35.5% of medial dislocations.

The duration of immobilisation remains a matter of debate. In the case of uncomplicated dislocations, it is often possible to put weight on the joint after a period of immobilisation lasting three-four weeks. For complicated dislocations, this period is frequently extended to six-eight weeks, depending on the type of associated fractures.

The literature describes a worse prognosis for lateral dislocations compared to medial forms [2] and an unsatisfactory long-term outcome for 10%–32% of lateral dislocations treated conservatively. The only long-term complication described for dislocations without associated fractures or injuries is a limitation of the ROM of the subtalar joint, with consequent difficulty in maintaining balance on uneven surfaces.

On the other hand, the incidence of infections and avascular necrosis is more than 30% in patients with lateral subtalar dislocations complicated by associated fractures or injuries of the soft tissues with or without contamination [57].

Indeed, lateral dislocations are more frequently associated with bone exposure and associated injuries. Furthermore, they more often require open reduction and the use of K-wires to guarantee the stability of the reduction.

In our review, we have deliberately not considered and tabulated the outcomes for the patients screened. In fact, different types of scores have been used in the articles examined to quantify the outcome and often the individual articles have grouped together types of patients who are very different from each other in clinical terms. In our opinion a comparison of the long-term outcome among these groups of patients would not be valuable (for example: outcome for patients with subtalar dislocation associated with bone exposure vs outcome for patients with subtalar dislocation without associated injuries). In four of the studies examined [16, 35, 37, 45], the AOFAS [58] scale was used, but this score has not been validated for this type of injury. Lastly, in many of the articles reviewed, no score has been used to quantify the outcome, but instead a qualitative description has been provided on the basis of the clinical course, making it impossible to compare them with the outcomes of other cases present in the literature.

Our review confirms the greater complexity of managing lateral dislocations compared to medial ones and reports the major pitfalls that must be taken into consideration by the surgeon dealing with this injury.
